# The Role of the MAPK Signaling, Topoisomerase and Dietary Bioactives in Controlling Cancer Incidence

**DOI:** 10.3390/diseases5020013

**Published:** 2017-04-26

**Authors:** Khaled A. Selim, Hend Abdelrasoul, Mohamed Aboelmagd, Ahmed M. Tawila

**Affiliations:** 1 Interfaculty Institute of Microbiology and Infection Medicine, Eberhard Karls Universität Tübingen, 72076 Tübingen, Germany; 2 Pharmaceutical and Drug Industries Research Division, National Research Centre, 33-El-Bohouth St. (former El Tahrir St.), Dokki, P.O. Box 12622, Giza, Egypt; maahmed@olemiss.edu (M.A.); Pharmazone2007@yahoo.com (A.M.T.); 3 Genetic Engineering and Biotechnology Research Division, National Research Centre, 33-El-Bohouth St. (former El Tahrir St.), Dokki, P.O. Box 12622, Giza, Egypt; hend.abdelrasoul@uni-ulm.de; 4 Institute of Immunology, Ulm University, 89081 Ulm, Germany; 5 National Center for Natural Products Research, School of Pharmacy, University of Mississippi, Oxford, MS 38677, USA

**Keywords:** dietary antioxidants, MAPK kinases, MAPK phosphatases, pterocarpans, ROS, topoisomerase

## Abstract

Reactive oxygen species (ROS) are common products of mitochondrial oxidative phosphorylation, xenobiotics metabolism and are generated in response to several environmental stress conditions. Some of them play important biochemical roles in cellular signal transduction and gene transcription. On the other hand, ROS are known to be involved in a wide range of human diseases, including cancer. The excessive production of such ROS together with disruption of homeostasis detoxifying mechanisms can mediate a series of cellular oxidative stresses. The oxidative stress of redundant free radicals production can lead to oxidative denaturation of cellular macromolecules including proteins, lipids and DNA. Moreover, oxidative damage is one of the major causes of DNA mutations, replication errors and genomic abnormalities which result in either inhibition or induction of transcription, and end with the disturbance of signal transduction pathways. Among affected signaling pathways are redox-sensitive kinases. The stimulation of these kinases induces several transcription factors through the phosphorylation of their module proteins. The activation of such pathways induces proliferation and cellular transformation. A diet rich in antioxidant compounds has potential health benefits, and there is a growing interest in the role of natural antioxidants in nutrition for prevention and cure of cancer diseases. A controversy has risen regarding the relation between antioxidants and the significant decrease in the risk of cancer incidence. In this review, we will focus on redox-sensitive kinases signaling pathways, highlighting the effects of dietary antioxidant on the prevention, incidence, prognosis or even treatment of human cancers. In addition, we will place emphasis on the chemical classes of pterocarpans as natural anti-oxidants/cancers as well as their underlying mechanisms of action, including their effects on MAPKs and topoisomerase activities.

## 1. Introduction

Reactive oxygen species (ROS) are known to be involved in a wide range of human diseases, including cancer. The oxidative damage that ROS cause can lead to DNA mutations which result in (either) (i) transcriptional inhibition or induction; (ii) enhancement of signal transduction pathways; (iii) replication errors or (iv) genomic abnormalities. ROS can be defined as a heterogeneous group of molecules that are, along with endogenous antioxidants, ubiquitously present. They are produced mainly in the aerobic cells. Mature myeloid cells generate ROS during innate immune responses. Low levels of ROS regulate normal cell proliferation as well as cell signaling [1]. Therefore, they act as a secondary messenger signaling molecules [2]. The harmful effect of oxygen arises when the tight regulation between the ROS and antioxidants is disturbed in a process called oxidative stress. This disturbance may occur due to excessive accumulation of ROS, shortage of antioxidant small molecules production, impaired antioxidant enzyme systems or altered transcriptional factors related to redox balance. 

## 2. Disturbance in ROS Levels Causes Diseases

**Figure 1:**
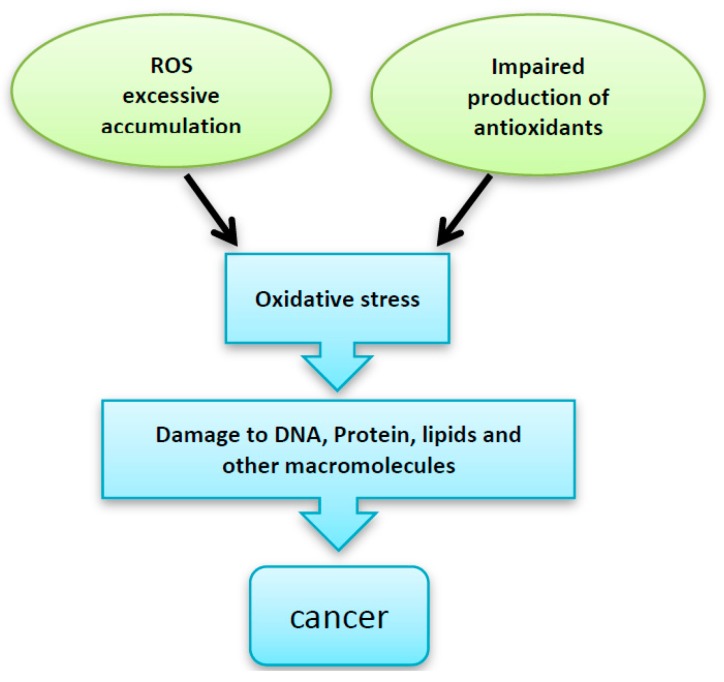
Excessive accumulation of reactive oxygen species (ROS), impaired production of the antioxidant enzymes and/or -impaired antioxidant defense system can be implicated in the DNA, protein and other intracellular macromolecules damage, which finally leads to malignant transformation.

Oxidative stress has been shown to be implicated in various clinical complications including malignant transformations [2]. ROS can cause DNA and protein oxidative damage, somatic mutations to tumor suppressor genes (such asP53). ROS can also induce the expression of proto-oncogenes [3]. Moreover, oxidative stress can have an effect on the signaling pathways of redox-sensitive kinases, e.g., Src, PI3K-Akt and MAPK (Erk, JNK, p38). These kinases regulate several transcription factors through the phosphorylation of their protein modules. Activation of such pathways can induce proliferation and cellular transformation [1,4] (Figure 1).

## 3. ROS and Cell Signaling 

**Figure 2:**
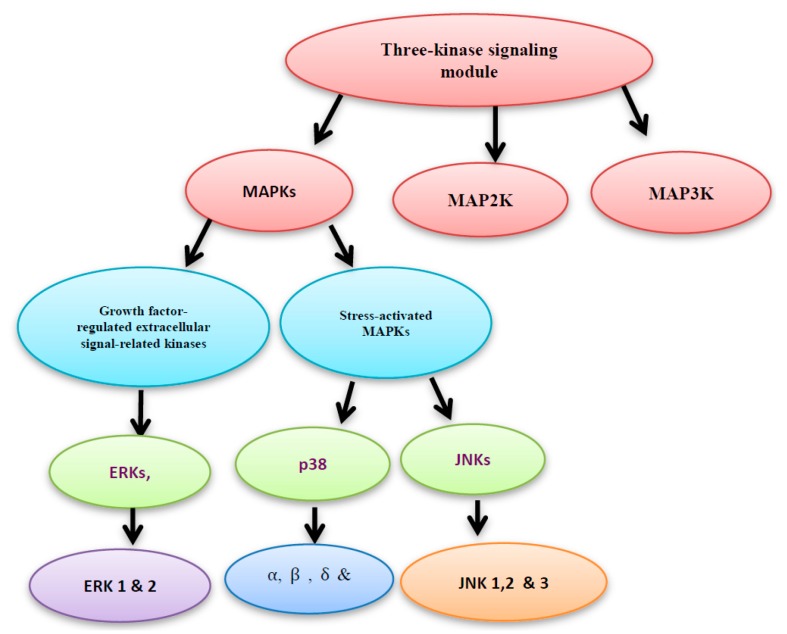
Mitogen activated protein kinase **(**MAPK), MAP2K and MAP3K construct the three- kinase signaling module system.

Two main protein families are known to regulate the ROS-activated signal transduction pathways, namely: the mitogen activated protein kinase (MAPK) and the redox sensitive kinases. Here, we will shed more light on the MAPK. The three-kinase signaling module system consists of MAPK, MAP2K and MAP3K. MAPKs include the growth factor-regulated extracellular signal-related kinases (ERKs), which are subdivided into ERK1 and 2 isoforms, and the stress-activated MAPKs. The stress-activated MAPKs can be categorized into c-jun NH2-terminal kinases (JNKs), which are subdivided into JNK1, 2 and 3 isoforms, and p38 MAPKs, which are subdivided into α, β, γ, and δ isoforms (Figure 2) [4].

## 4. MAPK Activation by MAP Kinases 

MAP Kinases (MAPKs) are a family of dual-specificity serine/threonine protein kinases, which mediate the transduction of extracellular signals from the cell membrane to the nucleus. They are also implicated in multiple cellular functions, ranging from cell survival and proliferation, to cell differentiation and programmed cell death [5]. After being activated via extracellular stimuli, MAPKs phosphorylate their substrates at serine and/or threonine residues. Such reversible phosphorylation can either enhance or inhibit the substrate; therefore, the entire signaling cascade activity can be changed [5]. 

**Figure 3:**
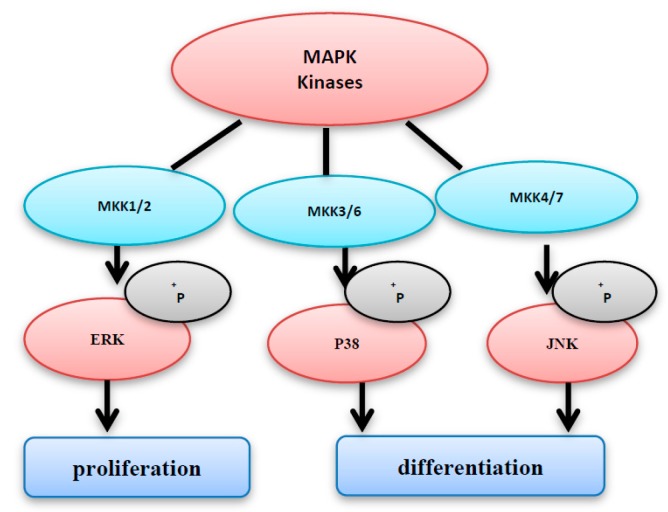
The MAPK kinases family consists of MKK1/2, MKK3/6, and MKK4/7. MKK1/2 and MKK3/6 activate ERK and p38, respectively. However, c-jun NH2-terminal kinase (JNK) is activated by MKK4/7 activation of extracellular signal-related kinase (ERK) enhances cell proliferation, however the activation of JNK and p38 induces cell differentiation and cell cycle arrest.

The MAP kinases family includes three members: MKK1/2, MKK3/6, and MKK4/7. ERK and p38 are activated by MKK1/2, and MKK3/6, respectively (Figure 3). However, JNK is activated by MKK4/7 [6,7,8]. After activation, MAPKs phosphorylate several substrates leading to the activation of diverse signal pathways, including proliferation, differentiation, and cell cycle arrest. It has been shown that activation of ERK enhances cell proliferation; however, the activation of JNK induces cell differentiation and cell death [8]. On the other hand, P38 activation does not necessarily promote cell death; instead, it is important for cell survival. This varying action may be dependent on the types of the stimuli and cells [9]. The three subgroups of MAPKs (i.e., ERKs, JNKs, and p38 MAPKs) play an important role in both cell growth and apoptosis (Figure 3), so that the tight orchestration of these pathways has a direct participation in the cell fate determination [4,10]. The unregulated activation of MAPK pathways may cause excessive production of MAPK-regulated genes; the expression of such genes will lead to abnormal proliferation, and unscheduled cell death.

## 5. MAPK Inhibition by MAPK Phosphatases

**Figure 4:**
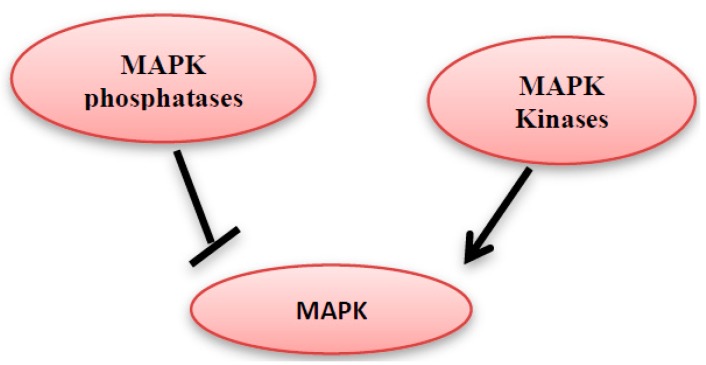
MAPK kinases can activate the MAPK, however they can be inhibited by MAPK phosphatases.

MAP kinase phosphatases (MKPs) are a large family of dual-specificity phosphatases [11]. They catalyze the dephosphorylation of threonine and/or tyrosine within the conserved TXY sequence of the MAP kinases and hence MAP kinase deactivation (Figure 4) [12,13]. The MKPs family members share the same sequence homology and affinity for MAPK proteins, but they differ in their substrate specificity, subcellular location, tissue distribution, and the induction by extracellular stimulants [12].

MKPs contain two main domains, namely a kinase-binding domain and a phosphatase domain. In the absence of a specific substrate, MKPs show low phosphatase activity. However, the enzymatic activity appears after binding to a specific MAPK in a process called substrate-induced activation mechanism. These two domains play an important role in the tight regulation of substrate specificity and enzymatic activity of the (MKPs) [11]. Several studies have showed that the oxidative stress can induce not only the MAPK kinases but also the MAPK phosphatases [8,14,15].

MKPs have high affinity to MAPKs but they have different substrate specificity for the MAPK family members. The MKP family can be subdivided into three groups [11,12,16,17]; Type I, Type II, and Type III. Type I MKP is a group of MKPs which is located in the nucleus and are activated by several stimuli, which, at the same time, can activate MAPKs. Therefore, this group is thought to play a vital role in the control of MAPK signaling in the nucleus. Type I MKPs include MKP-1, MKP-2, PAC1, and hVH3 [12].

Several studies classified MKP-1 as a stress-activated gene [14,15], while others referred to it as an ERK-specific phosphatase [18,19].Later, some studies showed that, in the case of oxidative stress, it can act on JNK and p38 [15,20]. Because of the ability of JNK, p38, and ERK to induce either differentiation or proliferation, the activation of MKP-1 should have a main role in the cell cycle regulation [21,22] or apoptosis [23,24]. Type II MKP, also called the Pyst subfamily, are dual-specificity cytoplasmic phosphatases. They can be subdivided into MKP-3, MKP-X and MKP-4, which are also called Pyst-1, 2 and 3, respectively, but they have nuclear export signal (NES). Restricted tissue distribution is a main feature of the members belonging to this group [8]. Finally, MKP-5, MKP-7 and M3/6 construct type III MKP can dephosphorylate JNK and p38, respectively but cannot dephosphorylate ERK1/2.

## 6. ROS Activate MAPK Pathway

Because of the special function of the MAPKs, as a mediator for both mitogen- and stress-activated signals, there is a growing interest to study the effect of ROS on these pathways. MAPK pathways can be activated at cellular levels by either extracellular or intracellular stimuli [4]. Several reports refer to the role of ROS in the MAPK pathways induction or mediation [25]. The activation of MAPK pathways by ROS at cellular levels can be proved by several evidences. For example, the intracellular signals that contribute in ROS production can also efficiently induce MAPK in different cell types. Moreover, the antioxidant enzymes which prevent the accumulation of ROS can also inhibit MAPK after cellular stimulation. Besides, the exogenous addition of an ROS like H_2_O_2_ enhances MAPK pathways, which indicates the role of ROS in the MAPK activation [4].

The role for ROS-activated Erk1/2 signaling in cell proliferation and malignant transformation is well established [26]. H_2_O_2_, which is generated as a byproduct during the estrogen metabolism in human breast cancer cells, activates Erk1/2, ultimately leading to cell proliferation enhancement. The inhibition of GDP/GTP exchange can also be involved in the MAPK/Erk1/2 activation by ROS after activation of Ras, which is an upstream activator for Erk1/2 through the activation of its cysteine 118 residues. Such activation can disturb the exchange between GDP and GTP Moreover, p90RSK, which is an upstream kinase of Erk1/2, can also be activated by ROS [26]. Furthermore, it has been shown that in ovarian cancer, the high levels of ROS are usually found to be combined with the absence of endogenous MKP3 which finally leads to activation of Erk1/2 and induction of cell proliferation as shown in Figure 3 [27].

Additionally, several reports indicated that the activation of Erk1/2 by ROS leads to induction of the cell survival and proliferation in different tumor types such as melanoma, breast cancer, ovarian cancer and leukemia [27]. Among the important factors that affect the activation of MAPK by ROS are the level of antioxidants reservoir and the cellular redox state; other factors are ROS concentration, site of production and accumulation. The mechanism for this activation is still not completely clear, but it is thought to include some oxidative aberrations of MAPK signaling proteins (e.g., RTKs and MAP3Ks) and/or inhibition of MKPs [4].

ROS have been detected in almost all cancers indicating a main role of ROS in tumor initiation and progression [2]. Intriguingly, the same cells produce elevated levels of antioxidants to detoxify ROS, suggesting that a tight balance between ROS and antioxidants is also required in order to keep the cells in a malignant state [27].

Ito et al. (2006) showed that the induction of p38 MAPK responding to the elevated levels of ROS decreases the life span of the Hematopoietic stem cells (HSCs) [28]. *Atm^−/−^* mice were used to prove that the accumulation of ROS enhances p38 MAPK phosphorylation which is HSC-specific and is associated with maintenance defect of HSC quiescence. However, the inhibition of p38 MAPK or long term treatment with antioxidant can rescue the cells and restore the population capacity. This indicates that, during the oxidative stress, ROS, particularly H_2_O_2_, can act as a second messenger to activate the p38 MAPK pathway which involved in exhaustion of the stem cells [28].

Recently, an important role of ROS-activated JNK as a regulator for p53 proapoptotic properties in cancer cells was identified. The study revealed how the p53 can switch the cell fate from growth arrest to apoptosis when it is pharmacologically activated in combination with thioredoxin reductase inhibition. Based on the fact that the malignant cells have limited ability to deal with high ROS levels, they suggest that elevated ROS, which resulted from such pharmaceutical treatment, creates an additional positive feedback loop for p53 through JNK activation. Such activation in mouse models might lead to a selective elimination of cancer cells by the restored p53 [3,28].

Sato et al. (2014) investigated the role of ROS in stem-like glioma-initiating cells (stem-like GICs) [28]. They found that the loss of self-renewal capacity and the induction of differentiation of stem-like GICs is mediated by ROS-activated p38 MAPK, which is activated by oxidative stress [27,29,30,31,32]. Interestingly, they also found that the ROS–activated p38 play a main role in FOXO3 activation and Bmi1 protein degradation during transition phase from the undifferentiated to a differentiated state as the activation of FOXO3 activate the cellular differentiation. However, the Bmi1 degradation leads to the loss of the self-renewal ability. Furthermore, oxidative stress can block the tumor-initiating capacity of GICs by activating the ROS-p38 cascade. They suggested that the ROS-activated p38 may be a novel node for therapeutic targeting of stem-like GICs. The dietary antioxidants prevent cancer incidence.

**Figure 5:**
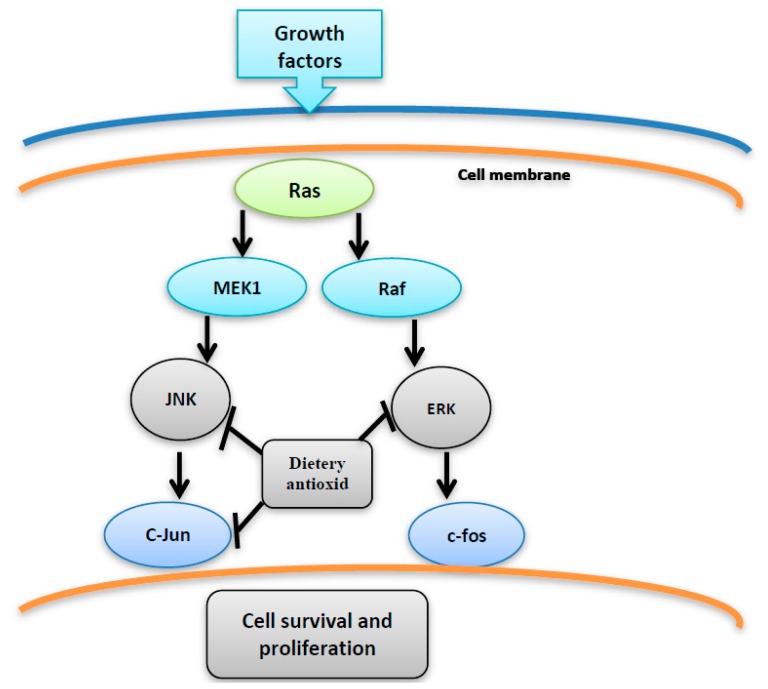
The inhibition of JNK, C-jun and ERK by dietary antioxidant resulting in suppression of cell proliferation and survival.

Several studies showed that appropriate antioxidant rich-diet could rescue nearly 30% of all cancer deaths in the United States by decreasing the oxidative stress that plays a main role in the development of many diseases, including cancer [2]. Accumulating research evidence refers to the effect of many dietary antioxidant elements which can be used alone or combined with traditional chemotherapy to prevent cancer occurrence or even to treat it. The main advantage of the natural antioxidants is the ability to reduce the cancer incidence risk by using such relatively nontoxic elements like fruits and vegetables, suggesting that the accumulation of antioxidant elements from these natural sources can create a chemopreventive effect without increasing the toxicity levels [2] (Figure 5).

Sheweita and Sheikh (2011) found a reverse relationship between the carcinogenesis grades and the antioxidant levels [33]. After histopathological examination of brain tumor samples, it has been noticed that the survival rate of the Grade III malignant glioma patients increased greatly by using specific doses of vitamin E (9.5 to 42.1 mg per day) [33,34]. Moreover, they observed a remarkable reduction in the incidence rate of such tumors in children whose mothers used these vitamins during their pregnancy periods. Furthermore, low antioxidant levels and considerable amounts of free radicals have been found to be combined with increased severity of brain tumors. Therefore, the antioxidants may have anti-carcinogenesis properties due to their function as free radical scavengers, telomerase inhibitors or as inhibitors for the nitrosation process [33].

Several studies involving apoptosis induced by vitamin E succinate in human MDA-MB-435 breast and SGC-7901 gastric cancer cell lines revealed the possible involvement of JNK, ERK1/2 pathways in addition to induction of NAG-1 expression in P38 kinase-dependent mechanism [35,36,37]. Conversely, some studies showed that the over-consumption of antioxidants may accelerate the tumor development. Sayin et al. (2014) have recently investigated the effect of adding antioxidants like *N*-acetylcysteine (NAC) and vitamin E to the diet of mouse models which were genetically engineered to develop lung tumors [26]. The per-weight dose was similar to the normal human use of them as a supplement. They detected a direct relation between the addition and the induction of the tumor progression and also reduction in the survival rate of these mice [26]. The deep sequencing of RNA showed an important change in the transcriptome profiles of the malignant cells. The presence of NAC and vitamin E together participated in the down-regulation of the endogenous antioxidant genes expression. It has also been shown that they can increase the proliferation of the lung tumor cells in both humans and mice by reducing the p53 expression, ROS and DNA damage [26]. Finally, it has been reported that the high risk population such as smokers or patients with chronic lung diseases should not consume antioxidants such as beta carotein because they may increase the growth rate of the tumor cells or implicate in more progression of precancerous injury [26].

Interestingly, green tea polyphenols (GT-polyphenols) have been shown to have an inhibitory effect on the skin cancer initiation in mouse models which were chemically-induced for skin cancer [38]. The effect of the GT-polyphenols has been tested by the topical application before using either the complete or the two-stage skin tumorigensis protocols. The effective inhibition may be due to the effect of epigallocatechin-3-gallate (EGCG),which is one of green tea polyphenols, on blocking the interaction between polycyclic aromatic hydrocarbons and epidermal DNA [2]. It is also known that, in hairless mice, either the oral consumption or topical application of brewed green tea, green tea extracts, or GT-polyphenols can cause high protection against UV or chemical-induced carcinogenesis [39]. Furthermore, the intraperitoneal injection of GT-polyphenols resulted in inhibition of the UV-induced skin papillomas’ growth rate [40]. Another study reported that the oral administration of green tea, black tea, or EGCG suppresses skin tumors’ growth rate [41]. Remarkably, in vivo experiments showed that the EGCG may act as a preventive agent against liver cancer, as it reduces the incidence of hepatoma in mice as well as the average number of hepatomas in each mouse [42].

Upon studying the cell growth and inhibitory effects of EGCG on human breast cancer cell line T47Da significant dose-dependent growth inhibition was observed after treatment. The study also revealed that there was an increase of phosphorylated JNK/SAPK protein until 24 h after administration; but, then it decreased. The phosphorylation of p38 protein was increased at 12 h and began to decrease at 36 h after catechin administration. The phosphorylated JNK/SAPK and p38 inhibited the phosphorylation of cdc2 and regulated the expression of cyclin A, cyclin B1, and cdk proteins, thus resulting in G2 arrest [43]. 

Additionally, Curcumin which is widely used as a spice was also found to enhance apoptosis in eight different melanoma cell lines, four of which have wild type P53 and the rest have the mutant form. Curcumin has been reported to be anti-oxidant, anti-cancer and antimicrobial agent. It acts also as a scavenger for a wide range of ROS [44]. Dorai et al. (2000) showed that Curcumin treatment enhances cell death in both androgen-dependent and androgen-independent prostate cancer cells. The cells were accompanied with downregulation of the proteins which inhibit apoptosis and other proteins like the androgen receptor [45].

Moreover, there are many epidemiological studies that correlate the dietary intake of flavonoids and isoflavonoids with the decrease of cancer incidence [46]. Genistein is one of the common isoflavonoids, which is found in many edible plants, and shows anti-carcinogenic effect in both animals and humans [47]. Genistein was found to cause cell growth inhibition in both of H460 cells and H322 cells, which have wild type p53 and the mutated form, respectively. The study showed that genistein treatment leads to up-regulation of the endogenous wild-type p53, while the level of the remaining mutant p53 protein did not change [48].

## 7. Topoisomerase

Topoisomerases are a group of essential enzymes that affect DNA topology through the relaxation of the supercoiling occurs during DNA replication, transcription and chromosomal condensation and segregation through nucleophilic attack of the phosphodiester bond forming a new ester bond between DNA and active tyrosine (Y723) residue of the enzyme allowing controlled rotation of the broken DNA strand. After relaxation, topoisomerase induces relegation of DNA, restoring the integrity of the DNA duplex [49]. 

The covalent intermediate formed through binding of topoisomerase and the DNA is called topoisomerase cleavage complex (TopCC). This complex is so transient and untraceable because ligation process of DNA is so much faster than cleavage. However, in the presence of certain inhibitors and/or DNA misaligning, TopCC can be trapped, resulting in apoptosis [49].

### 7.1. Classification of Topoisomerases 

Topoisomerases are divided into two types depending on the number of strands they cut.

A type I topoisomerase cuts one strand of a DNA double helix, relaxation occurs, and then the cut strand is re-ligated. Cutting one strand allows the part of the molecule on one side of the cut to rotate around the uncut strand, thereby reducing stress.

Type I topoisomerases do not require ATP for hydrolysis, they subdivided into three subclasses:

Type IA topoisomerases, which form a covalent intermediate with the 5′ end of DNA.

Type IB topoisomerases, which form a covalent intermediate with the 3′ end of DNA.

Type IC topoisomerase (also called Topoisomerase V) has been identified. While it is structurally unique from type IA and IB topoisomerases, it shares a similar mechanism with type IB topoisomerase.

A type II topoisomerase cuts both strands of one DNA double helix, passes another unbroken DNA helix through it, and then re-ligates the cut strands. Type II topoisomerases utilize ATP hydrolysis and are subdivided into two subclasses which possess similar structure and mechanisms:

Type IIA topoisomerases which include eukaryotic and eukaryal viral Topoisomerase IIα and Topoisomerase IIβ, bacterial gyrase, and topoisomerase IV.

Type IIB topoisomerases, which include Topoisomerase VI found in archaea.

### 7.2. Topoisomerase Inhibition 

Topoisomerase inhibitors are divided into two types:

Topoisomerase I inhibitors: irinotecan, topotecan, camptothecin and lamellarin D all target type IB topoisomerases,

Topoisomerase II inhibitors: etoposide, teniposide, doxorubicin, daunorubicin, mitoxantrone, amsacrine, ellipticines, aurintricarboxylic acid and HU-331, a quinolone synthesized from cannabidiol.

Certain natural phenols (ex. EGCG, resveratrol) showed marked inhibitory activity on both types of enzymes [50,51]. Recently, these inhibitors are believed to act as potent chemotherapeutic agents against cancer. Topoisomerase inhibition is also the mechanism by which flouroquinolone derivatives such as ciprofloxacin exert their antibacterial activity [52].

## 8. Pterocarpans as Anti-Oxidant/Cancer Agents

**Figure 6:**
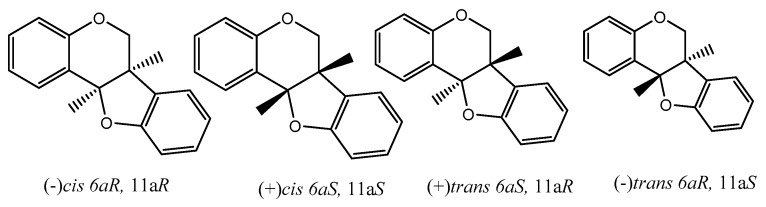
Four possible isomers of pterocarpans.

There are many reviews dealing with the anti-oxidant/cancer activity of flavonoids and generally with special concern of isoflavonoids. From here onwards, we would like to dedicate some attention to pterocarpans, a special class of flavonoid compounds which found in many plant families. Pterocarpans are found mainly in various species belonging to edible plants of family Leguminosae. Although they possess a capable anticancer activity with different mechanisms such that pterocarpans are a promising group of anticancer moieties, more focus on their activity is needed. Additionally, pterocarpans are considered the second largest group of naturally isoflavonoids. The core skeleton consists of a tetracyclic system with benzofuran-benzopyran fused rings, and the stereochemistry of the pterocarpan molecule determined by the two chiral centers at position 6a, 11a results in four possible isomers: two *cis*, (−)-(6a*S*, 11a*S*), (+)-(6a*R*, 11a*R*) and two trans, (−)-(6a*S*, 11a*R*), (+)-(6a*R*, 11a*S*) (Figure 6).

**Figure 7:**
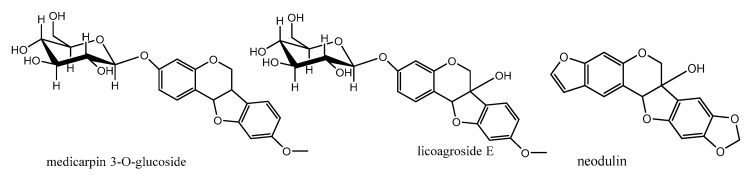
Different pterocarpans classes.

Pterocarpans were divided into two main classes (6a,11a-dihydro-6*H*-benzofuro[3,2-c]chromene) which is the main skeleton of pterocarpans and (6a,11a-dihydro-6*H*-benzofuro[3,2-c]chromene-6a-ol) or 6a hydroxypterocarpans. Goel and coworkers (2012) classified these two main classes of pterocarpans into three subclasses [53], as following:

The first subclass is *O*-Glycosylated pterocarpans: in these compounds the sugar part is mainly glucose or galactose and it is more common in pterocarpans with the main skeleton ex. medicarpin than in the 6a hydroxypterocarpans ex. licoagroside E (Figure 7). The second subclass is dimethylpyranopterocarpans: which are biologically active moieties formed by enzyme catalyzed isoprenylation usually in **3** hydroxy and/or **9** hydroxy positions followed by interamolecular cyclization in the available adjacent positions. ex. neorautenol (compound **18**).The third class is furanopterocarpans: in this subclass furan ring is fused to pterocarpan molecule comparable to pyranopterocarpans these compounds are rarely seen in nature, ex. neodulin (Figure 7).

**Figure 8:**
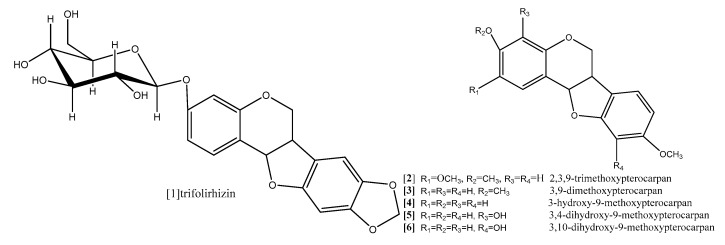
9-Methoxypterocarpans derivatives.

Zhoh and coworkers (2009) reported that pterocarpan trifolirhizin (compound **1**; Figure 8) which was isolated from roots of *Sophora flavescens,* (Leguminosae) exhibited antiproliferative activity in dose-dependent manner against human(A2780) ovarian and (H23) lung cancer cells lines after 24 h incubation [54]. The antiproliferative activity done by MTT assay was not observed with concentrations less than 50 μM, while significant antiproliferative effect was detected with concentrations up to 100 μM in human (A2780) ovarian, and with concentration up to 250 μM for (H23) lung [54]. It was also reported in another study of morphological changes observed with epifluorescence microscope after 3 days that trifolirhizin suppressed human myeloid leukemia (HL-60) through induction of apoptosis [55]. Trifolirhizin was also reported to induce apoptosis in MKN45 cancer cells. The mechanism by which trifolirhizin induced apoptosis in vitro and in vivo was mediated via EGFR-MAPK pathways. Western blotting was used to investigate the levels of apoptotic and related signaling pathway proteins [56].

Herein, this study trifolirhizin also showed dose-dependent suppression of LPS-stimulated TNF-α, IL-6, and COX-2 in mouse macrophages, it could be concluded that trifolirhizin, which belongs to *O*-Glycosylated pterocarpans subclass, exhibited its anticancer activity through more than one mechanism, induction apoptosis, inhibition of proliferation, and suppression of inflammatory mediators which play a critical role in the process of tumorigensis. Aggarwal and coworker (2006) reported that chronic inflammation can lead to cancer and the inflammatory mediators as TNF, interleukins, chemokines, COX-2, 5-LOX, and MMP-9 are involved in many steps of the process of tumorigensis [57] which is consistent with many other studies that show a clear link between the anti-inflammatory properties of flavonoids and their anticancer activity [58].

The anticancer and the mode of action of 9-methoxypterocarpans derivatives were studied tentatively by Militao and coworkers in series of studies [59,60,61]. They reported that these classes of pterocarpans possess strong cytotoxic activity against different human cancer cell lines. 2,3,9-trimethoxypterocarpan (compound **2**; Figure 8), 3,9-dimethoxypterocarpan (Homocarpin) (compound **3**; Figure 8), 3-hydroxy,9-methoxypterocarpan (Medicarpin) (compound **4**; Figure 8), and 3,4-dihydroxy,9-methoxypterocarpan (Vesticarpin) (compound **5**; Figure 8), were tested against five human cancer cell lines including two leukemic cell lines (CEM) and (HL-60), colon adenocarcinoma (HCT-8), breast adenocarcinoma (MCF-7), and murine melanoma (B-16). Cell viability was determined by the trypan blue dye exclusion test and IC_50_ values were estimated after 3, 6, 12, 24, 36, 48, 60 and 72 h of incubation. The results revealed that compound **3** is more cytotoxic than compound **5** in the three tested cell lines (CEM), (HL-60) and (HCT-8) with IC_50_ values (5.5, 3.9, 6.4 and 7.3, 6.9, 12.4 µg/mL) respectively. Compound **3** showed a two-fold increase in activity in (HL-60) and (HCT-8) cell lines in comparison with compound **5**. Compound **2** has potent cytotoxicity against all tested cell lines with IC_50_ values (0.6, 0.1, 0.7, 0.6 and 2.9 µg/mL) respectively, and it was considered a promising cytotoxic molecule. It could be concluded that methoxy substitution on C-2 in homocarpin and compound **2** are responsible for stronger cytotoxicity [62].

Moreover, the mode of action of medicarpin (compound **4**; Figure 8) was studied on myeloid leukemia cells TRAIL-induced apoptosis at a dose of 20 μM. The result revealed the possible JNK activation involvement [63].

Continuing their study for the anticancer effect of 9-methoxypterocarpans derivatives, Militao and coworkers [55] conducted antimitotic study for compounds **2**–**6** (Figure 8), which were isolated from *Platymiscium floribundum* heart wood. The pterocarpans showed dose-dependent antimitotic activity, the alteration effect of the tested compounds on the egg development in this assay gives an idea of the mechanism of cytotoxicity which could be related to DNA, protein inhibition and/or inhibition of microtubules assembly. All tested pterocarpans showed strong antimitotic activity, with IC_50_ values very close to that of reference drugs doxorubicin and etoposide. Compound **2** was about 1000 times more active than the two reference drugs. Clearly, these results confirm the importance of methoxy substitution on C-2 in the pterocarpan moiety, the methoxy group on C-9 is common feature in all of the active pterocarpans, replacement of methoxy group at C-3 with OH gives comparable activity, but increasing the ratio of OH to methoxy group in the pterocarpan molecule decreases the antimitotic activity [64].

Moreover, the same author, Militao et al. (2006), explored the ability of 9-methoxypterocarpans derivatives to induce apoptosis as part of their cytotoxicity using human promyleocytic leukemia (HL-60) cell line [60]. The results showed that all tested pterocarpans significantly caused DNA fragmentation and inhibited the DNA synthesis at concentration 12.5 µg/mL and also induced activation of caspase-3 in HL-60 cells. Both compounds **4** and **5** were able to disrupt mitochondrial cell membrane integrity in a concentration-dependent manner. Additionally, the cytotoxicity study revealed a reduction in the number of viable cells with an increased number of non-viable cells, while the methoxylated analogue compounds **2** and **3** caused no damage to the cell membrane and at the same time reduced the number of viable cells without increasing the number of non-viable cells. Besides, the cell cycle analysis showed that both compounds **2** and **3** arrested the cell cycle at G2/M phase at concentration 12 µg/mL. It was concluded clearly that all tested pterocarpans exhibited common apoptotic features and an emphasis on pterocarpans with more hydroxyl substitutions seems to induce necrosis, while apoptosis is more liable with methoxylated pterocarpans [60].

In a comparative study the antiproliferative effect of compound **2** was evaluated by the trypan blue dye exclusion test against panel of four leukemic cell lines JURKAT, HL60, K562 and MOLT-4, the IC_50_ values were 8–18.8 µM these results indicated that compound **2** exhibited its effect in both a dose- and time-dependent manner where significant results were obtained after 48 h and 72 h with IC_50_ values of 0.3 to 2.5 µM, 0.3 to1.6 µM respectively [59].

In recent in-depth study, Militao et al. (2014) studied the anticancer mechanism of compound **2** on the cell cycle progress and microtubule function in three breast cancer cell lines MCF7, T47d and HS578T [65]. The cell cycle arrest was induced in the all tested cell lines by compound **2** at concentration 8 µM in a time-dependent manner where a 24 h incubation period followed by a 24 h recovery period in medium free pterocarpan led to a reversible effect while persistent mitotic inhibition followed by apoptosis was noticed after a 48 h exposure period despite the pterocarpan free medium recovery period. In this study, the mitosis was inhibited during prometaphase, in a crucial step where the separation of duplicated centrosomes was blocked followed by cell cycle arrest, and the persistent prometaphase arrest resulted in apoptosis [65]. 

**Figure 9:**
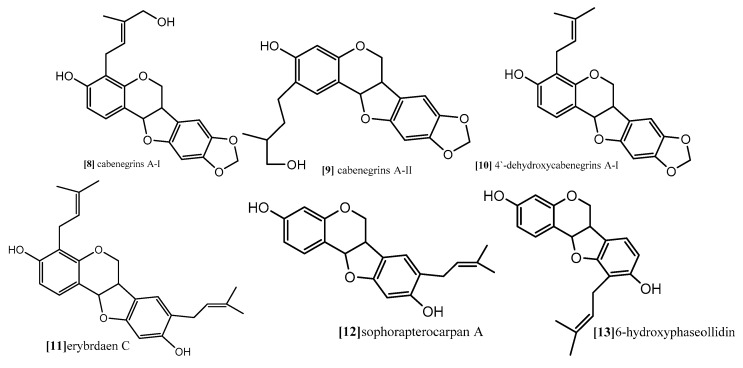
Examples of prenylatedpterocarpans. Topoisomerase inhibition as a promising target for cancer treatment.

**Figure 10:**
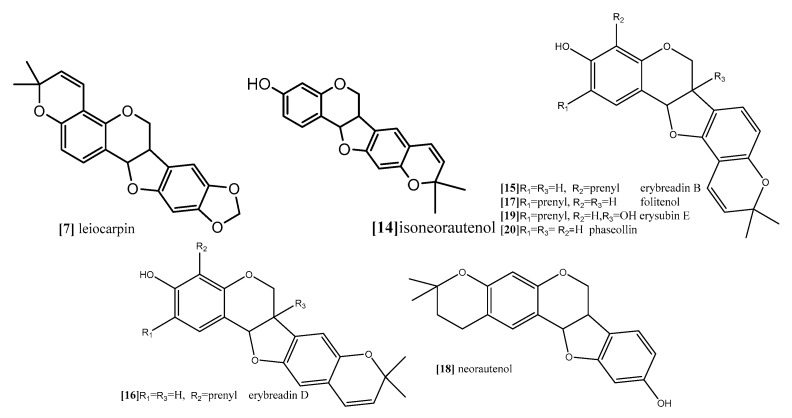
Examples of pyranopterocarpans.

The same antimitotic assay was employed by other authors in another study to evaluate the antimitotic effect of prenylatedpterocarpans cabenegrinsA-I (compound **8**; Figure 9), cabenegrins A-II (compound **9**; Figure 9), 4′-dehydroxycabenegrin A-I (compound **10**; Figure 9) and leiocarpin (compound **7**; Figure 10). The results also indicate that neither the hydroxyl group on C-3 nor the methylenedioxy moiety on C-8, C-9 are essential pharmacophoric units for the antimitotic activity, while the presence of prenyl group at position C-2, C-4 increases the antimitotic activity [61].

As mentioned before, both topoisomerases I and II are responsible for cleavage of DNA. While topoisomerase I is able to cleave one DNA strand, topoisomerase II cleaves both DNA strands. However, their function is essential for almost all DNA metabolism processes, and tyrosine (Tyr^723^) is the binding site where topoisomerases start their nucleophilic attack and form DNA cleavable complex [49,66]. Erybraedin C, a prenylatedpterocarpan (compound **11**; Figure 9) which was isolated from *Bituminaria bituminosa* flower (Leguminosae) showed antitumor activity against two human adenocarcinoma cell lines (HT29 and LoVo) proficient and deficient in MMR (mismatch repair), p53 and Bcl-2 [66]. In a recent study, the molecular mechanism exploring the antitumor activity of compound **11** revealed that it demonstrates its effect through inhibition of topoisomerase I function but in a manner different from the other common topoisomerase I inhibitors. Usually, topoisomerase inhibitors are divided into catalytic or posions. The posions inhibitors bind reversibly to the enzyme-DNA complex after the step of DNA cleavage and delay the DNA relegation step, which induces cell death. The catalytic inhibitors, on the other hand, interfere with one of other steps of the topoisomerase I cycle. Compound **11** exhibited irreversible biding to DNA-enzyme complex, thus completely inhibiting the relegation step and also binding to the topoisomerase I itself. The molecular modeling study revealed that the preferential binding site of compound **11** on the enzyme come within proximity of tyrosine (Tyr^723^) active site, and its prenyl substitution on C-8 position come in close contact with two active sites residue, (Arg^488^) and (His^632^). These are involved in the catalytic reaction of the enzyme on the DNA strand, inhibiting their function so that it blocks the DNA cleavage step. Another interesting finding from the modeling study is the binding of compound **11** to the enzyme which gave enough space fitting for the DNA substrate into the enzyme cavity, so that compound **11** is considered the first example of a natural compound able to inhibit topoisomerase I reaction in both the cleavage and relegation steps without inhibition of the enzyme binding [66].

Kuete and coworker (2014) provided a mechanistic study for the antiproliferative effect of two pterocarpans Sophora pterocarpan A and 6α-hydroxyphaseollidin which were isolated from the bark of *Erythrina sigmoidea* (Leguminosae) in one study [67], and for one pterocarpan isoneorautenol which was isolated from roots of *Erythrina excelsa* (Leguminosae) in another study [68]. The cytotoxic activity of these pterocarpans was evaluated against panel of nine sensitive and resistance cell lines, drug-sensitive (CCRF-CEM) and multidrug-resistant P-glycoprotein (P-gp) over expressing (CEM/ADR5000) leukemia, the (MDA-MB-231-pcDNA3) breast cancer and its resistant subline (MDA-MB-231-BCRP clone 23)(breast cancer resistance protein clone 23), the (HCT116) (p53^+/+^) colon cancer cells and its knockout clones (HCT116) (p53^−/−^), the (U87MG) glioblastoma cells and its resistant subline epidermal growth factor receptor (U87MG. ΔEGFR) and human hepatocellular carcinoma cells (HepG2) and the normal hepatocytes (AML12). Both pterocarpans Sophorapterocarpan (compound **12**; Figure 9) and 6α-hydroxyphaseollidin(compound **13**; Figure 9) exhibited strong cytotoxic effects against all tested cancer cell lines with IC_50_ values 3.6 to 6.4 µM and 3.7 to 14.8 µM, respectively, also isoneorautenol(compound **14**; Figure 10) showed cytotoxicity against the nine tested cell lines with IC_50_ values below 22 µM. In the same study, the cell cycle analysis of the two pterocarpans compounds **12** and **13** against leukemia (CCRF-CEM) cells demonstrated that both of them induced cell cycle arrest in the Go/G1 phase. Compound **14** induced cell cycle arrest between Go/G1 phase and S phase in a time-dependent manner. It has been proven from the results that the tested pterocarpans induces apoptosis in leukemia (CCRF-CEM) cells via different modes of action. Compound **13** was able to increase the activity of caspases which are responsible for cutting the cellular proteins at specific Aspartate residues to regulate the process of apoptosis, compound **13** activated the initiator caspase 3/7 and effectors caspases 8 and 9 two-fold in concentration range (0.5–2-fold IC_50_), while compound **14** activated caspases 3/7 more than caspases 8 and 9 at a concentration of 2-fold IC_50_. In another method, compound **12** induces apoptosis through the breakdown of mitochondrial membrane potential (MMP) the disruption of which is a common event in the process of apoptosis by (17%–92.9%) in concentration range form (1/4-fold IC_50_ to 2-fold IC_50_). Similarly, compound **14** displayed dose-dependent disruption of MMP (89% at concentration of 2-fold IC_50_).

Furthermore, Nguyen and coworkers (2009) analyzed the cytotoxic activity of 15 isolated pterocarpans from stem bark of *Erythrina abyssinica* (Leguminosae) against the panel of four breast cancer cell lines including drug resistance types [69]. The results of cytotoxicity indicated that erybraedin B (compound **15**; Figure 10) is the most active compound with IC_50_ values (5.6 to 11.8 µM), it exhibited 2-fold activity of tamoxifen (the reference drug) against the drug-resistant cell lines, tamoxifen-resistant MCF7 (MCF7/TAMR) and adriamycin-resistant MCF7 (MCF7/ADR) cell lines with IC_50_ (6.2 ± 0.2 and 5.6 ± 0.7 µM), respectively. It has been proven that the presence of 2,2-dimethypyran substitution in ring D of the pterocarpan molecule is the most important feature of the cytotoxic activity against breast cancer cell lines where the presence of this moiety in erybraedin B (compound **15**; Figure 10), erybraedin D (compound **16**; Figure 10), and folitenol (compound **17**; Figure 10) demonstrated potent cytotoxicity that exceeded that of compounds which lacked this moiety. Moreover, it was concluded from this study that there is strong correlation between the cytotoxicity against the breast cancer cell lines and the inhibition of protein tyrosine phosphatase 1B (PTP1B) activity where pterocarpans that showed strong cytotoxicity exhibited potent inhibition of PTP1B activity. Prenylation of the pterocarpan molecules appear to be important pharmacophoric features for both inhibition of PTP1B and cytotoxic effect on breast cancer cell lines, while the absence of this moiety is accompanied with diminished activity in both assay systems. Prenylation at position C-4 in the tested pterocarpans seemed to be important requirement for the potent inhibition of PTP1B activity, where compounds **15**, **16** and **11** share the same feature and showed IC_50_ of 4.2, 6.4, 7.3 µM respectively. On the other hand, pterocarpans with prenylation at C-2 showed lower inhibitory effect, whereas neorautenol (compound **18**; Figure 10), folitenol (compound **17**; Figure 10) and erysubin E (compound **19**; Figure 10) showed IC_50_ values of 7.6, 7.8, 8.8 µM, respectively. The interesting point here is that hydroxylation of C-6 of compound **18** diminished its cytotoxicity but kept its inhibition property of PTP1B. It could be concluded that the inhibition of PTP1B is supposed to be related to breast carcinogenesis inhibition, and the selective inhibition of PTP1B emerges as new strategy for the treatment of breast cancer [69]. The genotoxicity of compound **18** and phaseollin (compound **20**; Figure 10) was further evaluated in terms of the ability to break the DNA strands in a study conducted by Wätjen and coworker (2007) against (HAII4) rat hepatoma cell line [70]. The results revealed that compounds **18** and **20** possess potent cytotoxicity with EC_50_ 1, 1.5 µM respectively. The analysis of their mechanism of cytotoxicity indicates that both compounds **18**, and **20** significantly increased the activity of caspase 3/7 enzymes, at concentration 1, 2 µM respectively, and the amount of fragmented nuclei also increased, which are signs of apoptosis. Also, it was found that both compounds **18** and **20** disrupted the cell membrane of (HAII4) cells which also indicates the ability of induction necrosis. Furthermore, it was found that compound **18** significantly breaks DNA strands while compound **20** showed no activity [70]. Additionally, all previous anticancer activities of pterocarpans compounds were summarized in Table 1.

## 9. Conclusions 

A controversy has arisen regarding the relation between antioxidants and the significant decrease in the risk of cancer incidence. Additionally, potent flavonoid-pterocarpans were proven to be potent and promising moieties for inhibition of cancer incidence. However, excessive screenings of the effects of dietary anti-oxidant/cancer pterocarpans on the prevention, incidence, and prognosis, or even the treatment of human cancers are still needed.

## Figures and Tables

**Table 1:** Summary of anticancer activities of pterocarpans assayed with various methods.

Compound	Method	Type of Cells	Incubation Period	Results	Ref.
Trifolirhizin	MTT	The A2780 ovarian cancer and H23 lung cancer cells	24 h	Significant antiproliferation was achieved with concentrations up to 100 μM against A2780 ovarian cancer cells. However, a significant antiproliferative effect was observed only with a concentration of 250 μM for H23 lung cancer cells.	[54]
Trifolirhizin	Morphological changes was observed with epifluorescence microscope	Human myeloid leukemia (HL-60)	3 days	Trifolirhizin suppressed human myeloid leukemia (HL-60) through induction of apoptosis	[55]
Trifolirhizin	MTT assay for cell viability. Hoechst 33342 staining and TUNEL staining for detection of apoptosis. Western blotting was used to investigate the levels of apoptotic and related signaling pathway proteins.	MKN45, L02, HEK293 cells	2 days	A concentration- and time-dependent suppression of MKN45 cell viability with IC_50_ 33.27 ± 2.06 μg/mL was observed. The apoptosis was mediated via EGFR-MAPK pathways. Trifolirhizin also arrested the G/M cycle through impact on Cdc2/cyclin B complex.	[56]
2,3,9-trimethoxypterocarpan, Homocarpin, Medicarpin and Vesticarpin	MTT	B16 (murine melanoma), HCT-8 (human colon), MCF-7 (human breast), CEM and HL-60	3, 6, 12, 24, 36, 48, 60, and 72 h	2,3,9-trimethoxypterocarpan was the most active compound against all human cancer cell lines with IC_50_ 2.9, 0.6, 0.7, 0.6, 0.1 μg/mL, respectively.	[62]
Medicarpin	Determination of cell viability and LDH Release Cell cycle and cell death analysis Measurement of ROS and the mitochondrial ROS Real-time PCR Cloning of the DR5 promoter and luciferase assay staining with phycoerythrin-conjugated mouse monoclonal anti-human DR5 or DR4 for analysis of cell surface expression of DR4 and DR5	The cell lines K562, LAMA-84 (chronic myeloid leukemia cell lines), U937, OCIAML-3 (the AML cell lines)	48 h	A trail-induced apoptosis at a dose 20 μM was observed. The result revealed the possibility of involvement of JNK activation.	[63]
2,3,9-trimethoxypterocarpan	The Trypan blue dye exclusion test	HL-60, K562, Jurkat, and Molt-4	3, 6, 12, 24, 36, 48, 60 and 72 h	After 24 h, Jurkat and Molt-4 showed less sensitivity (IC_50_ > 10 and 5.9 ± 1.1 g/mL, respectively) while HL-60 (IC_50_ 2.5 ± 0.3 g/mL) and K562 cells showed (IC_50_ 2.8 ± 0.67 g/mL). After 36 h, the IC_50_ values ranged from 0.5 to 1.1 g/mL, without significant difference among the cell lines. Maximum activity was observed after 48 h of incubation, with K562 being the most resistant cell line (IC_50_ 0.8 ± 0.1 g/mL), followed by Molt-4 and HL-60 (both with IC_50_ of 0.4 g/mL), and Jurkat (IC_50_ 0.1 ± 0.03 g/mL).	[59]
2,3,9-trimethoxypterocarpan	Cell cycle analysis and measurement of the mitochondrial transmembrane potential	Breast cancer cell lines MCF7, T47d and HS578T	24 and 48 h	The cell cycle arrest was induced in the all tested cell lines at concentration 8 µM in time-dependent manner where 24 h incubation period followed by 24 h recovery period in medium free pterocarpan led to a reversible effect while persistent mitotic inhibition followed by apoptosis was noticed after a 48 h exposure period despite the pterocarpan free medium recovery period. Mitosis was also inhibited during the prometa phase, in a crucial step where the separation of duplicated centrosomes was blocked followed by cell cycle arrest, and the persistent prometaphase arrest resulted in apoptosis of treatment, the IC_50_ values ranged from 0.3 to 1.6 mM	[65]
Erybraedin C and bitucarpin A	Hemocytometer cell count	HT29 and LoVo human colon adenocarcinoma	LoVo, 26 h and HT29, 29 h	Erybraedin C and bitucarpin A induced antitumor activity against two human adenocarcinoma cell line (HT29 and LoVo) proficient and deficient in MMR (mismatch repair), p53 and Bcl-2	[66]
Sophorapterocarpan A, 6α-hydroxyphaseollidin	resazurin reduction assay Flow cytometry for cell cycle analysis Analysis of mitochondrial membrane potential (MMP)	panel of nine sensitive and resistance cell lines, drug-sensitive (CCRF-CEM) and multidrug-resistant P-glycoprotein (P-gp) over expressing (CEM/ADR5000) leukemia, the (MDA-MB-231-pcDNA3) breast cancer and its resistant subline (MDA-MB- 231-BCRP)(breast cancer resistance protein clone 23), the (HCT116) (p53^+/+^) colon cancer cells and its knockout clones (HCT116) (p53^−/−^), the (U87MG) glioblastoma cells and its resistant subline epidermal growth factor receptor (U87MG. ΔEGFR) and human hepatocellular carcinoma cells (HepG2) and the normal hepatocytes (AML12).	48 and 72 h	pterocarpansSophorapterocarpan (compound **12**; Figure 9) and 6α-hydroxyphaseollidin(compound **13**; Figure 9) exhibited strong cytotoxic effects against all tested cancer cell lines with IC_50_ values 3.6 to 6.4 µM and 3.7 to 14.8 µM, respectively. In the same study the cell cycle analysis of the two pterocarpans compounds **12** and **13** against leukemia (CCRF-CEM) cells demonstrated that both of them induced cell cycle arrest in the Go/G1 phase. Compound **13** was able to increase the activity of caspases which are responsible for cutting the cellular proteins at specific Aspartate residues to regulate the process of apoptosis, compound **13** activated the initiator caspase 3/7 and effectors caspases 8 and 9 two-fold in concentration range (0.5–2 fold IC_50_) In another method, compound **12** induces apoptosis through breakdown of mitochondrial membrane potential (MMP) the disruption of which is a common event in the process of apoptosis by (17%–92.9%) in concentration range form (1/4-fold IC_50_ to 2-fold IC_50_).	[67]
Isoneorautenol	Resazurin reduction assay Flow cytometry for cell cycle analysis Analysis of mitochondrial membrane potential (MMP)	panel of nine sensitive and resistance cell lines, drug-sensitive (CCRF-CEM) and multidrug-resistant P-glycoprotein (P-gp) over expressing (CEM/ADR5000) leukemia, the (MDA-MB-231-pcDNA3) breast cancer and its resistant subline (MDA-MB- 231-BCRP) (breast cancer resistance protein clone 23), the (HCT116) (p53^+/+^) colon cancer cells and its knockout clones (HCT116) (p53^−/−^), the (U87MG) glioblastoma cells and its resistant subline epidermal growth factor receptor (U87MG. ΔEGFR) and human hepatocellular carcinoma cells (HepG2) and the normal hepatocytes (AML12).	48 and 72 h	Isoneorautenol (compound **14**; Figure 10) showed cytotoxicity against the nine tested cell lines with IC_50_ values below 22 µM. Compound **14** induced cell cycle arrest between Go/G1 phase and S phase in time-dependent manner. It has been proven from the results that the tested pterocarpans induces apoptosis in leukemia (CCRF-CEM) cells via different mode of action. It also activated caspases 3/7 more than caspases 8 and 9 at a concentration of 2-fold IC50. It displayed dose-dependent disruption of MMP (89% at concentration of 2-fold IC_50_) as well.	[68]
Erybraedin B, erybraedin D, folitenol, neorautenol and erysubin E	The cell viability was assessed using a 4-[3-(4-iodophenyl)-2-(4-nitrophenyl)-2*H*-5-tetrazolio]-1,3-benzene disulfonate (WST-1) based cytotoxicity assay Inhibitory effects on protein tyrosine phosphatase-1B (PTP1B)	MCF7, tamoxifen-resistant MCF7 (MCF7/TAMR), adriamycin-resistant MCF7 (MCF7/ADR) and MDA-MB-231 breast cancer cell lines.		Erybraedin B (compound **15**; Figure 10) is the most active compound with IC_50_ values (5.6 to 11.8 µM), it exhibited 2-fold activity of tamoxifen the reference drug against the drug resistance cell lines, tamoxifen-resistant MCF7 (MCF7/TAMR) and adriamycin-resistant MCF7 (MCF7/ADR) cell lines with IC_50_ (6.2 ± 0.2 and 5.6 ± 0.7 µM), respectively. It has been proven that the presence of 2,2 dimethypyran substitution in ring D of the pterocarpan molecule is the most important feature of the cytotoxic activity against breast cancer cell lines where the presence of this moiety in erybraedin B (compound **15**; Figure 10), erybraedin D(compound **16**; Figure 10), folitenol (compound **17**; Figure 10) demonstrated potent cytotoxicity more than compound which lack this moiety. Moreover, it was concluded from this study that there is strong correlation between the cytotoxicity against the breast cancer cell lines and the inhibition of protein tyrosine phosphatase 1B (PTP1B) activity where pterocarpans that showed strong cytotoxicity exhibited potent inhibition to PTP1B activity. Prenylation of the pterocarpan molecules appear to be important pharmacophoric feature for both inhibition of PTP 1B and cytotoxic effect on breast cancer cell lines, while the absence of this moiety is accompanied with diminished activity in both assay systems. Prenylation at position C-4 in the tested pterocarpans seemed to be an important requirement for the potent inhibition of PTP1B activity, where compounds **15**, **16** and **11** share the same feature and showed IC_50_ (4.2, 6.4, 7.3 µM) respectively. On the other hand, pterocarpans with prenylation at C-2 showed lower inhibitory effect, whereas neorautenol (compound **18**; Figure 10), folitenol (compound **17**; Figure 10) and erysubin E (compound **19**; Figure 10) showed IC_50_ values of 7.6, 7.8, 8.8 µM, respectively. The interesting point here is hydroxylation of C-6 of compound **18** diminishes its cytotoxicity but keeps its inhibition property of PTP1B. It could be concluded that the inhibition of PTP1B is supposed to be related to breast carcinogenesis inhibition, and the selective inhibition of PTP1B is emerging as a new strategy for the treatment of breast cancer	[69]
Neorautenol and phaseollin		(HAII4) rat hepatoma cell line		Both compounds possess potent cytotoxicity with EC_50_ 1, 1.5 µM respectively. The analysis of their mechanism of cytotoxicity indicates that both compounds significantly increased the activity of caspase 3/7 enzymes, at concentration of 1, 2 µM respectively, and the amount of fragmented nuclei also increased, which are signs of apoptosis. Also, it was found that both compounds disrupted the cell membrane of (HAII4) cells which also indicates the ability of induction necrosis. Furthermore, it was found that Neorautenol significantly breaks DNA strands while phaseollin showed no activity	[70]
